# Satisfaction with the Aesthetic Effect and Quality of Life for Women after Breast Conserving Therapy (BCT)—Preliminary Research

**DOI:** 10.3390/ijerph16234682

**Published:** 2019-11-25

**Authors:** Agata Krzos, Andrzej Stanisławek, Marian Jędrych, Marta Łuczyk, Barbara Ślusarska

**Affiliations:** 1 Department of Oncology, Chair of Oncology and Environmental Health, Medical University of Lublin, S. Staszica 4-6 St., 20-081 Lublin, Poland; andrzej.stanislawek@umlub.pl; 2 Department of Medical Informatics and Statistics with E-learning Lab, Medical University of Lublin, K. Jaczewskiego 4 St., 20-090 Lublin, Poland; marian.jedrych@umlub.pl; 3 Department of Family Medicine and Community Nursing, Chair of Oncology and Environmental Health, Medical University of Lublin, S. Staszica 4-6 St., 20-081 Lublin, Poland; barbara.slusarska@umlub.pl

**Keywords:** breast cancer, breast-conserving surgery, patient satisfaction, quality of life, Breast-Q

## Abstract

All methods of breast cancer treatment may potentially lead to breast deformities, which are often associated with the reduced mental well-being of patients. Breast conserving therapy (BCT) is commonly used, and its core element is breast conserving surgery (BCS).The aim of this study was to determine the level of satisfaction with the aesthetic outcome of surgery and quality of life (QoL) of breast cancer patients undergoing BCT in a longitudinal study performed three months, six months and 12 months after surgery. This longitudinal observational study was carried out on a group of 91 women. The Breast-Q^TM^ BCT 1.0 questionnaire was used in this study. Before surgery, patients assessed their satisfaction with the appearance of their breasts (SwB) at an average level of M = 56.0. Satisfaction with the aesthetic outcome (SwO) of BCS was highest among all patients three months after surgery (M = 63.0). The mean score in the sub-scale of psychosocial well-being (Psycho-soc W-B) before surgery was M = 62.0, while in the subsequent periods of the study, it was higher. The mean score for physical well-being (Physical W-B) before surgery was M = 69.92; and in the subsequent study periods, it was lower. The level of patient satisfaction with the outcome of the surgery and the QoL related to health do not differ significantly in post-operative observation. QoL in terms of psychosocial functioning in patients undergoing BCT is significantly higher 12 months after surgery compared to the pre-operative period. Patient satisfaction with the BCS aesthetic outcome is positively related to the evaluation of QoL in terms of psychosocial functioning.

## 1. Introduction

In the Polish population, breast cancer is the most common malignant cancer in women. It is also the second most common cause of cancer deaths (after lung cancer) among women. Epidemiological data from 2016 indicate that in Poland there were 18,615 cases of breast cancer and the standardized incidence rate was 54.1/100,000 [1]. The most recent data published in 2019 by Globocan indicate that the global breast cancer incidence rate in 2018 was 46.3/100,000, with the highest in Australia and New Zealand (94.2/100,000) and the lowest in South Central Asia (25.9/1,000,000) [2].

Breast conserving therapy (BCT) includes breast conserving surgery (BCS), which is followed by radiotherapy to eradicate the disease. BCS involving the removal of a tumor with adjacent healthy tissue and/or axillary lymph nodes, combined with radiotherapy, is associated with long-term results—in terms of local recurrence, disease-free interval and overall survival—similar to those in the case of mastectomy [3,4,5]. Comprehensive oncological treatment in the form of surgical intervention combined with chemotherapy, radiotherapy or hormone therapy increases the chances of women with breast cancer being cured. At the same time, the specificity of breast cancer itself and the therapeutic actions taken have a particular impact on the somatic and mental states of women. All methods of breast cancer treatment may potentially lead to breast deformities. This situation is often associated with a reduction in the mental well-being of patients [6,7,8,9,10,11]. Although BCT is generally considered less mutilating than breast amputation, attention should be given to the problems of women undergoing this form of treatment, and emotional and psychosocial support should be provided. While research on the quality of life (QoL) related to the health of women undergoing surgery for breast cancer and the psychosocial consequences of breast surgery has a long history, research on satisfaction with the aesthetic effect and its impact on women’s lives is a topic that is only now beginning to be addressed [12,13,14]. The use of standardized assessment tools will allow for an exchange of patients’ opinions in the international context [15].

Literature shows that factors influencing the effect of BCS are classified into two categories: those referring to the patient (e.g., breast size, body weight, age, body mass index (BMI)) and those concerning the tumor and treatment (e.g., tumor size and site, specimen weight or volume, surgical technique, radiotherapy, chemotherapy) [6,7].

One element of subjective evaluation of the aesthetic outcome of BCT is the patient’s self-assessment. This is probably the simplest measure for analyzing aesthetic effects following BCT. Undoubtedly, self-esteem carries over into patients’ psychosocial adaptation to the outcome of the surgery. The main argument for the legitimacy of applying this method is the fact that it is the patient who must live and cope with treatment outcomes. A crucial aspect that needs to be taken into account is the fact that patients may inflate their assessments intentionally so as not to offend their attending doctors, particularly when questionnaires are given to patients during follow-up visits or in the form of emails provided by their doctors. Another essential issue that should be taken into consideration is the way patients regard BCT. For them, it is an alternative to mastectomy so even if results are not fully satisfactory, it is assessed higher than breast amputation [7]. In Poland, there have been few reliable studies of the problem concerning assessment of the aesthetic effect and women’s satisfaction with BCT.

The aim of this study was to determine the level of satisfaction with the aesthetic outcome of surgery and QoL of breast cancer patients undergoing BCT in a longitudinal study performed three months, six months and 12 months after the operation. The relationship between patient satisfaction with the aesthetic effect of BCS and the QoL associated with health was also investigated.

## 2. Materials and Methods 

### 2.1. Study Design

**Figure 1:**
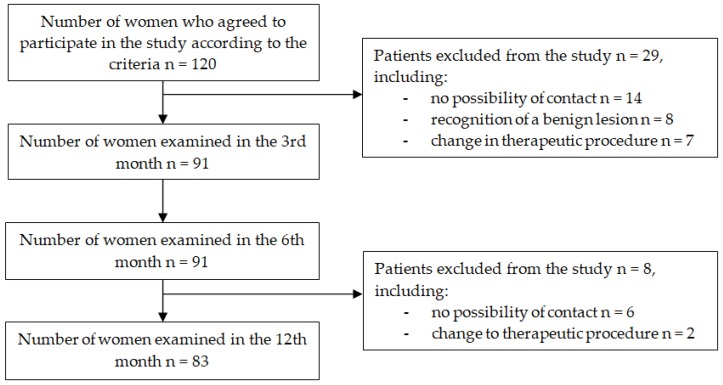
Procedure for women’s qualification for the study.

This longitudinal observational study was carried out in a group of women with breast cancer undergoing BCT. The selection of participants was a non-random sample of women reporting for treatment to the Oncology Center. The inclusion criteria were as follows: malignant breast cancer in histopathological examination, conserving unilateral surgery, obtaining informed consent of the patient before surgery, and lack of consciousness disorders, dementia or mental disease in the women. Before surgery, 120 patients had agreed to participate in the study. The research analysis included material collected from 91 patients, and at the last stage of the study, in the 12th month after surgery, only 83 patients were included in the study. A diagram of the procedure for recruiting the group of patients is presented in Figure 1.

### 2.2. Participants

Recruitment of women for the study was conducted among patients who had undergone BCS in one of the reference centers for comprehensive treatment of malignant cancers in the region of Lublin, Poland. The study was conducted from May 2015 to February 2018.

The first meetings with patients in the pre-operative period were held in the Oncological Surgery Department of the Oncology Center one day before surgery. At this stage, the patients were acquainted with the course of the study, received written information about the project, and signed an informed consent to participate in the study. They were also asked to complete the pre-operative module of the Breast-Q questionnaire together with a questionnaire compiled by the authors only for this study. Subsequent meetings with patients in the 3rd, 6th and 12th month after their surgery were held during follow-up visits in the Oncological Outpatient Clinic of the Oncology Center. At these stages, the patients were asked to complete the post-operative module of the Breast-Q questionnaire with personal information.

### 2.3. Ethical Approval

The consent of the Bioethics Committee at the Medical University of Lublin to conduct the research project was obtained (Resolution of the Bioethics Committee No. KE-0254/240/2014).

### 2.4. Questionnaire

The Breast-Q^TM^ BCT 1.0 questionnaire) was used in the study by Pusic, Cano & Klassen (Memorial Sloan Kattering Cancer Center and The University of British Colombia) [15,16]. Appropriate consent was obtained to use the Polish version of the tool in 2014. The validation of the tool into Polish, accepted by the owners of the tool, was carried out by Basta (Jagiellonian University in Krakow).

The Breast-Q is a newly-developed tool for measuring the opinions of patients after BCT and plastic surgery that is utilized in the USA, Canada, Brazil, Australia, Asian countries including China and India, Israel, Saudi Arabia, and European countries such as the Netherlands, Spain, Germany, Great Britain, Italy, Sweden, Hungary, Norway, France and Ireland. The effectiveness of the tool has been confirmed in the literature [15,16]. The questionnaire consists of pre- and post-operative modules. The module to evaluate patient satisfaction with the pre-operative appearance of their breasts (SwB scale) included: perception of the appearance of the breasts and adjustment of underwear and clothing, while the satisfaction module with the aesthetic outcome of the surgery (SwO scale) examined the above parameters in the post-operative period. The tool is also used to measure the quality of life related to health in the following sub-scales: psychosocial well-being (Psych-soc W-B scale; aspects of emotional and social health); physical well-being (Physical W-B scale; experiencing discomfort and pain at the site of breast surgery); sexual well-being. The questionnaire also contains modules focusing on the measurement of satisfaction with care: satisfaction with information provided by the breast surgeon, information provided by the radiologist, assessment of the professionalism of the surgeon, assessment of the professionalism of the radiotherapist, satisfaction with the service provided by administrative staff members.

Each module contained subordinate questions and the “raw” result was transformed, according to the authors’ instructions, into a result on a scale from 0 to 100, where a higher score meant higher satisfaction and QoL. On the basis of our own research, the reliability of the Breast-Q questionnaire was estimated within the scope of sub-scale SwO, where the Alfa Cronbach scale value of 0.79 was obtained. The reliability of internal consistency using the Alfa value standardized for the calculation of the Alfa Cronbach value was 0.79. The analysis of the reliability of the Breast-Q questionnaire of the Psycho-soc W-B sub-scale after surgery was estimated by Alfa Cronbach at 0.81. The reliability of internal consistency using the standardized value of Alfa for the calculation of Alfa Cronbach was 0.82. The analysis of the reliability of the Breast-Q questionnaire for the Physical W-B sub-scale after the surgery for the calculation of Alfa Cronbach value was estimated at 0.87. The reliability of internal consistency using the standardized value of Alfa for the calculation of the Alfa Cronbach value was 0.87.

### 2.5. Statistical Analysis

The material collected was subjected to statistical analysis. The results of the study are summarized in tables calculating the percentages (%) in the statistical evaluation of qualitative features, while mean values (M), standard deviation (SD), minimum (Min), and maximum (Max) were calculated for quantitative features. Due to the fact that the traits being researched (in terms of SwB, SwO and Psych-soc W-B sub-scales) did not have a normal distribution, the main statistical analysis was performed using the Wilcoxon test to compare two samples of dependent variables and the ANOVA Friedman test to compare multiple samples of dependent variables. The examination of the relationship between the measured variables expressed on an orderly scale was based on the Spearman’s Rank Correlation Factor. The significance of the relationship between the tested traits was found at the level of *p* < 0.05. The study results were elaborated using the STATISTICA 13.3 (StatSoft Polska) program.

## 3. Results

### 3.1. Participant Characteristics

The mean age of the women researched was 56.6 years. The youngest patient was aged 29 and the oldest 72. Women under 50 years of age constituted 24.2% (*n* = 23) of the group examined, 42.9% (*n* = 39) were between 50 and 60 years of age, and 33.0% (*n* = 30) of all the examined women were over 61 years of age. In 57.1% of women, cancer was found in the right breast, no special type (NST) cancer was diagnosed most frequently (82.4%) in histopathological examination. Table 1 presents the socio-demographic and clinical data characterizing the research participants after BCS.

### 3.2. Results of Breast-Q Questionnaire

Before surgery, patients assessed their satisfaction with the appearance of their breasts (SwB) at an average level of M = 56.0, SD = 11.9 (Table 2). Satisfaction with the aesthetic effect of BCS (SwO) was highest among all the patients three months after the surgery (M = 63.0; SD = 13.2).

The score on the Psych-soc W-B sub-scale determined among 91 patients before surgery was M = 62.0 (SD = 14.2), while in subsequent periods of the study, it was higher (Table 2).

The mean QoL score was determined for the Physical W-B sub-scale using the Breast-Q questionnaire in the group of women researched (Table 2), which was M = 69.9 (SD = 8.6) before surgery; it was lower in the subsequent study periods.

Significant differences were found in the results for the SwB and SwO sub-scales obtained at the successive stages of pre- and post-operative testing [ANOVA Friedman, Chi^2^ ANOVA: (*N* = 83, the degrees of freedom (df) = 3) = 40.592; *p* < 0.001; Table 3]. Additionally, comparison of satisfaction levels before, and three months after, surgery (Wilcoxon test: *n* = 91; value of Wilcoxon test statistic (Z) = 4.026; *p* < 0.001) demonstrated an increase in satisfaction in the post-operative period. Similarly, higher satisfaction levels were observed at six months after surgery (Wilcoxon test: *n* = 91; Z = 3.699; *p* < 0.001) and 12 months after surgery (Wilcoxon test: *n* = 83; Z = 3.819; *p* < 0.001) compared to satisfaction with the appearance of the breasts before the surgery. It should be noted that comparison of satisfaction results among the three post-operative periods showed no differences (ANOVA Friedman, Chi^2^ANOVA: (*N* = 83, df = 2) = 0.591; *p* > 0.05).

Comparison of results on the Psycho-soc W-B sub-scale before and three months after the surgery (Wilcoxon test: *n* = 91; Z = 2.371; *p* < 0.05) showed an increase in satisfaction in the post-operative period. Similarly, a higher score on the Psych-soc W-B sub-scale was observed at the six-month time point (Wilcoxon test: *n* = 91; Z = 2.281; *p* < 0.05) and at 12 months after the surgery (Wilcoxon test: *n* = 83; Z = 3.437; *p* < 0.001) compared to pre-operative results. In comparing mean results obtained from patients on the Psych-soc W-B sub-scale in all observations (both before and in all post-operative periods), differences were found in subsequent stages of the study (ANOVA Friedman, Chi^2^ ANOVA: (*n* = 83, df = 3) = 17.214; *p* < 0.001; Table 3). The lowest mean results obtained on this sub-scale were in terms of QoL before the surgery, while the highest level of psychosocial functioning was recorded 12 months after the surgery. Comparison of mean results on the Psych-soc W-B sub-scale in the three post-operative periods of the study did not indicate any significant differences in QoL (Table 3).

There were no differences in the average results on the Physical W-B sub-scale obtained in successive stages of pre- and post-operative testing (Table 3). Similarly, a comparison of mean results on the Physical W-B sub-scale in the three post-operative periods of the study did not show any differences in the results obtained (*p* > 0.05). The highest mean score on the sub-scale was observed in the period before surgery (M = 70.4; SD = 8.6) and was only lower in subsequent stages of the study.

The data reveals a positive correlation between average results on the SwB and SwO sub-scales and the average result on the Psych-soc W-B sub-scale at all stages of the longitudinal observation (Table 4). Along with the increase in average results on the psychosocial sub-scale, an increase in patient satisfaction measured with the Breast-Q questionnaire can be observed.

There was no correlation between the average result on the SwB and SwO sub-scales and the average result on the Physical W-B sub-scale in either the pre- or post-operative periods. The results obtained are presented in Table 4.

In the next part of the analysis, the relationship between the average results on the Psych-soc W-B sub-scale and the average results on the Physical W-B sub-scale obtained at all stages of the study was evaluated (Table 4). A positive correlation was found between the results of the above-mentioned modules of the Breast-Q questionnaire obtained in the third month (*p* < 0.05) and sixth month after surgery (*p* < 0.05).

## 4. Discussion

Women’s satisfaction with the aesthetic outcome following BCS has not been a common topic in research analyses. To our knowledge, there are no scientific reports comparing the results of the Breast-Q questionnaire obtained in the pre- and post-operative periods in a group of women having undergone BCS without reconstructive surgery, which makes it impossible for us to have a broader discussion in this area.

The study by Vrouwe et al. [17] of women undergoing BCT conducted within three years following the surgery shows that the average score on the SwO sub-scale in the Breast-Q questionnaire was 59.3 (SD = 21.1) points and this is slightly lower than that obtained in the present study. In turn, in a study of 200 post-BCS patients, O’Connell et al. [18] determined the mean SwO score of patients to be 69.0 (SD = 20.0), which is significantly higher than in this study. Results similar to those obtained in this study are presented by Lagendijk et al., showing an SwO score of 65.7 points (SD = 22.4) on a group of 257 patients undergoing BCT [19].

Comparing the average results of the Breast-Q questionnaire in terms of Psycho-soc W-B and Physical W-B sub-scales obtained in this study with the results of other authors’ research, significant differences should be noted. Results similar to these obtained in this study, i.e., 70.1 points (SD = 21.4), have been obtained by researchers in the Netherlands [19]. On the other hand, in studies conducted among patients in Canada, significantly higher results have been obtained: 73.5 points (SD = 21.2) [17]. Similarly, high scores on the Psycho-soc W-B sub-scale (M = 78.0; SD = 22.0) have been achieved in a study among patients from the United Kingdom [18].

The result of 71.2 (SD = 18.9) [19] on the Physical W-B sub-scale obtained among the patients from the Netherlands is similar to the results of our study. A significantly higher score of 74.0 (SD = 19.1) was obtained among Canadian patients [17] and 76.0 (SD = 18.0) among UK patients [18].

Research by Kim et al. [20] on 64 women who had had quadrantectomies with partial reconstruction using the widest dorsal lobe muscle demonstrates satisfaction with the aesthetic effect and with the level of psychosocial and physical functioning by means of the Breast-Q questionnaire using a reconstruction module. The mean score on the SwO sub-scale is 82.4 (SD = 18.2) points, which is significantly higher than that in this study. Moreover, the average result on the Psycho-soc W-B sub-scale is 76.0 (SD = 16.7), and that on the Physical W-B sub-scale is 68.7 (SD = 16.5). 

The study by Flanagan et al. on a group of women who had undergone BCT and mastectomy with subsequent implant reconstruction proves that time after surgery is an important factor in breast satisfaction and QoL. Satisfaction with the aesthetic outcome after surgery decreases with time, regardless of the type of surgery, while psychosocial well-being improves [21]. Similar results have also been obtained by other authors [22]. In our study, no significant differences were observed in post-operative results of the Breast-Q questionnaire obtained at different time intervals; analysis of the mean results can indicate a similar trend.

Studies on the satisfaction of patients with the aesthetic effect of the operated breast, with a focus on determining physical and mental well-being, make it possible to see the problems experienced by the patient and to differentiate their severity in comparison with the pre-operative period. In our study, a significantly lower average result on the SwB sub-scale was found, compared to the result on the SwO sub-scale, after surgery. This may be associated with the psychological burden of a cancer diagnosis, uncertain prognosis, and necessity of surgery. The quality of life in terms of the Psycho-soc W-B sub-scale was observed to be significantly higher after surgery. Similar results are found by Głowacka et al., who researched 50 women who had undergone BCT with a sentinel node biopsy procedure. The EORTC QLQ-C30 (European Organization for Research and Treatment of Cancer Quality of Life Questionnaire-Core 30) and QLQ-BR 23 (Quality of Life Questionnaire - Breast Cancer Module) questionnaires demonstrate an improvement in emotional and psychological functioning after the surgery. In both the aforementioned research and our own research, the first stage of the study, carried out immediately before surgery, coincided with the start of anticancer treatment, a period associated with stress and feelings of uncertainty and risk. The lower intensity of emotional tension after surgery can be explained by the reduction of uncertainty concerning type of surgery and the exclusion of the need for mastectomy [8].

The present analysis was carried out on a relatively small group of women because of difficulties in obtaining patient consent to participate in long-term studies, or difficulties in contacting patients in the post-operative period. It does however appear that the results may constitute a point of reference in discussions with other authors raising the issue of satisfaction with the aesthetic outcome of women undergoing BCT. Another limitation of the results obtained is the fact that the majority of the study participants were women over 50 years of age, which may be associated with attributing less importance to the aesthetic effect of the breast by this age group.

One of the strengths of this study is the use of a specific tool to measure patient satisfaction with the aesthetic effect achieved, i.e. the Breast-Q tool, the reliability of which was confirmed in the study group.

## 5. Conclusions

1. The level of patient satisfaction with the outcome of the surgery and the QoL related to health (according to the Breast-Q) do not differ significantly at any stage of long-term post-operative follow-up.
2. The level of satisfaction with the aesthetic outcome of the surgery at all stages of post-operative assessment is higher than the level of satisfaction of women with their breast appearance before the operation.
3. The QoL in terms of psychosocial functioning in patients with breast cancer undergoing BCT is significantly higher 12 months after surgery than in the pre-operative period.
4. Patient satisfaction with the aesthetic outcome of the surgery has a strong positive relationship with the evaluation of their QoL in terms of psychosocial functioning at all stages of longitudinal observation.

## Figures and Tables

**Table 1:** Characteristics of the study group.

Socio-Demographic and Clinical Characteristics		%	*n*
Place of residence	country	30.8	28
	city<100 thousand citizens	41.8	38
	city>100 thousand citizens	27.4	25
Marital status	married	78.0	71
	single	4.4	4
	widowed	9.9	9
	divorced	5.5	5
	informal relationship	2.2	2
Education	primary	2.1	2
	vocational	20.9	19
	secondary	44.0	40
	higher	33.0	30
Hormonal status	post-menopausal	73.6	67
	pre-menopausal	26.4	24
Brest cancer	right	57.1	52
	left	42.9	39
Histological type of cancer	no special type (NST)	82.4	75
	invasive lobular carcinoma (ILC)	3.3	3
	ductal carcinoma in situ (DCIS)	7.7	7
	other (tubular, medullary, mucous)	6.6	6
Scope of surgical operations within regional lymph nodes	SLNB	83.5	76
	ALND	12.1	11
	no	4.4	4

NST—no special type cancer; ILC—invasive lobular carcinoma; DCIS—ductal carcinoma in situ; SLNB—sentinel lymph node biopsy; ALND—axillary lymph node dissection.

**Table 2:** Mean results of the Breast-Q questionnaire in individual observation periods

Breast-Q BCT Domain	Observation Period	M	SD	−95%	+95%	*n*	Min	Max
SwB	before operation	56.0	11.9	53.5	58.5	91	29	100
SwO	3 months after	63.0	13.2	60.2	65.7	91	27	100
	6 months after	61.8	11.0	59.5	64.1	91	43	100
	12 months after	62.2	12.7	59.4	64.9	83	41	100
Psych-soc W-B	before operation	62.0	14.2	59.0	65.0	91	34	100
	3 months after	66.2	17.2	62.6	69.8	91	32	100
	6 months after	65.9	15.4	62.7	69.1	91	35	100
	12 months after	68.4	16.5	64.8	72.0	83	36	100
Physical W-B	before operation	69.9	8.6	68.1	71.7	91	50	80
	3 months after	67.9	14.9	64.8	71.0	91	34	100
	6 months after	67.5	15.1	64.4	70.6	91	25	100
	12 months after	67.9	15.4	64.5	71.2	83	0	100

M—mean; SD—standard deviation; *n*—number; Min—minimum; Max—maximum; 95%confidence interval; BCT—breast conserving therapy; SwB—satisfaction with breast; SwO—satisfaction with outcome; Psycho-soc W-B—psychosocial well-being; Physical W-B—physical well-being

**Table 3:** Comparison of the results of Breast-Q questionnaire in individual observation periods (*n* = 83).

Breast-Q BCT Domain	Observation Period	M	SD	−95%	+95%	Min	Max	ANOVA Friedman	
								Chi ^2^	*p*
SwB	before operation	55.0	10.2	52.7	57.2	29	100	40.592 ^1^	<0.001 ^1^
SwO	3 months after	62.6	12.7	59.8	65.4	39	100		
	6 months after	61.4	11.1	59.0	63.8	43	100		
	12 months after	62.2	12.7	59.4	64.9	41	100	0.591 ^2^	>0.05 ^2^
Psych-soc W-B	before operation	61.6	13.8	58.6	64.6	34	93		
	3 months after	66.4	17.3	62.7	70.2	32	100	17.214 ^1^	<0.001 ^1^
	6 months after	66.4	15.1	63.1	69.7	35	100		
	12 months after	68.4	16.5	64.8	72.0	36	100	2.947^2^	>0.05 ^2^
Physical W-B	before operation	70.4	8.6	68.5	72.2	53	80		
	3 months after	69.2	14.5	66.1	72.4	34	100	1.630 ^1^	>0.05 ^1^
	6 months after	68.3	15.0	65.0	71.6	25	100		
	12 months after	67.9	15.4	64.5	71.2	0	100	1.047 ^2^	>0.05 ^2^

M—mean; SD—standard deviation; Min—minimum; Max—maximum; 95%—confidence interval; BCT—breast conserving therapy; SwB—satisfaction with breast; SwO—satisfaction with outcome; Psycho-soc W-B—psychosocial well-being; Physical W-B—physical well-being; ^1^ Comparison of Breast-Q questionnaire results before and 3 months, 6 months, and 12 months after surgery; ^2^ Comparison of Breast-Q questionnaire results 3 months, 6 months, and 12 months after surgery.

**Table 4:** Relationship between the results on the Breast-Q questionnaire subscales in research participants.

Breast-Q BCT Domain	Observation Period	*n*	R	T (*n*−2)	*p*
SwB vs. Psych-soc W-B	before operation	91	0.410	4.243	<0.001
SwO vs. Psych-soc W-B	3 months after	91	0.502	5.477	<0.001
	6 months after	91	0.447	4.721	<0.001
	12 months after	83	0.516	5.421	<0.001
SwB vs. Physical W-B	before operation	91	0.048	0.454	>0.05
SwO vs. Physical W-B	3 months after	91	0.097	0.916	>0.05
	6 months after	91	0.056	0.526	>0.05
	12 months after	83	0.064	0.574	>0.05
Psych-soc W-B vs. Physical W-B	before operation	91	−0.099	−0.937	>0.05
Psych-soc W-B vs. Physical W-B	3 months after	91	0.215	2.077	<0.05
	6 months after	91	0.236	2.294	<0.05
	12 months after	83	0.189	1.730	>0.05

n—number; R—Spearman’s Rank correlation coefficient; t (*n*−2)— Student’s t-test; *n*−2 = 2 degree of freedom are lost because two means are calculated one mean for each group whose means are to be composed; BCT – breast conserving therapy; SwB – satisfaction with breast; SwO – satisfaction with outcome; Psycho-soc W-B – psychosocial well-being; Physical W-B – physical well-being.
